# Hepatocytic transcriptional signatures predict comparative drug interaction potential of rifamycin antibiotics

**DOI:** 10.1038/s41598-020-69228-z

**Published:** 2020-07-28

**Authors:** Shetty Ravi Dyavar, Timothy M. Mykris, Lee C. Winchester, Kimberly K. Scarsi, Courtney V. Fletcher, Anthony T. Podany

**Affiliations:** Antiviral Pharmacology Laboratory, University of Nebraska Medical Center (UNMC) Center for Drug Discovery UNMC, Omaha, NE 68198 USA

**Keywords:** Pharmacology, Antimicrobials

## Abstract

Current strategies to treat tuberculosis (TB) and co-morbidities involve multidrug combination therapies. Rifamycin antibiotics are a key component of TB therapy and a common source of drug–drug interactions (DDIs) due to induction of drug metabolizing enzymes (DMEs). Management of rifamycin DDIs are complex, particularly in patients with co-morbidities, and differences in DDI potential between rifamycin antibiotics are not well established. DME profiles induced in response to tuberculosis antibiotics (rifampin, rifabutin and rifapentine) were compared in primary human hepatocytes. We identified rifamycin induced DMEs, cytochrome P450 (*CYP*) *2C8*/*3A4*/*3A5*, *SULT2A,* and *UGT1A4*/*1A5* and predicted lower DDIs of rifapentine with 58 clinical drugs used to treat co-morbidities in TB patients. Transcriptional networks and upstream regulator analyses showed *FOXA3*, *HNF4α*, *NR1I2*, *NR1I3*, *NR3C1* and *RXRα* as key transcriptional regulators of rifamycin induced DMEs. Our study findings are an important resource to design effective medication regimens to treat common co-conditions in TB patients.

## Introduction

Tuberculosis (TB) is a leading cause of morbidity and mortality worldwide^1^ and antibiotic therapy is a major infection-controlling measure in persons with either latent or active TB infection^2^. Human immunodeificency virus (HIV)-TB co-infected persons are susceptible to bacterial, viral, and fungal opportunistic infections (OIs) and moreover, cancer co-exist in more than four percent of active TB patients, which add to morbidity and mortality^3–8^. Concurrent treatment of TB, HIV, OIs and cancer often necessitates polypharmacy, and requires management of drug-drug interactions (DDI) to prevent detrimental treatment outcomes.

Drug-susceptible TB is currently treated with 6 months of daily rifampin therapy, which is combined with other antibiotics such as isoniazid, pyrazinamide, and ethambutol^9^. Additionally, many of the current treatment options for latent tuberculosis infection (LTBI) and prophylactic regimens for TB involve the use of rifampin or rifapentine^9–11^. Rifamycin antbiotics are known to be a common cause of DDIs with co-administered medications via induction of drug metabolizing enzymes (DMEs). Clinical DDI data guide the management of rifamycin DDIs during polypharmacy, but comprehensive clinical DDI data are not available across all rifamycin antibiotics to manage common medication therapies to treat co-morbidities such as HIV and OIs in TB patients^10,11^.

The primary human hepatocyte (PHH) model is a gold standard to unravel potential DDIs through expression profiling of genes encoding enzymes and transporters that alter drug metabolism^12^. In this process, the application of highly sensitive next generation sequencing (NGS) technology determines changes in extremely low DME transcript copy numbers expressed in PHHs to understand their role in metabolism and disposition of drugs interacting with rifamycins. Then, transcriptomic data obtained in NGS is used in a downstream systems pharmacology approach to identify novel targets, which allows better understanding of potential DDIs and prediction of effectiveness of a drug regimen during co-prescription of medications^13–16^.

Several potential DDIs associated with rifamycins remain unaddressed. First, studies utilizing NGS to profile rifamycin responsive genes (RGs) to predict clinical DDI outcomes have not been conducted. Second, data available on rifampin associated DDIs are complex due to in-vitro data generation in alternative hepatic cell line models and seldom in PHHs^17^. While translatability of these alternative hepatocyte models to PHHs is unclear, complexity is further increased when PHHs were derived from volunteers who were exposed to alcohol, tobacco, and medications which may have a direct influence on DME profiles and thereby potential DDI inferences^18,19^. Finally, no comparative study has been performed on transcriptional responses of rifampin, rifabutin, and rifapentine in PHHs to examine and compare independent and integrated gene signatures, underlying pathways, networks, and metabolic programs, which are key molecular events underlying rifamycin mediated DME expression and associated DDIs.

In response to these key knowledge gaps, we applied NGS in tandem with systems pharmacology tools to determine DME expression profiles in metabolically active PHHs derived from three healthy (drug/tobacco/alcohol free) volunteers in response to rifampin, rifabutin, and rifapentine. We have identified integrated transcriptional signatures of rifamycin drugs and pathways that regulate drug metabolism, networks of genes, and transcriptional programs regulating drug metabolism. This information was used to predict outcomes of potential DDIs with rifamycins and drugs used to treat HIV, cancer, and other common disease states in TB patients.

## Results

### Rapid metabolism of rifapentine in PHHs among rifamycins

**Figure 1:**
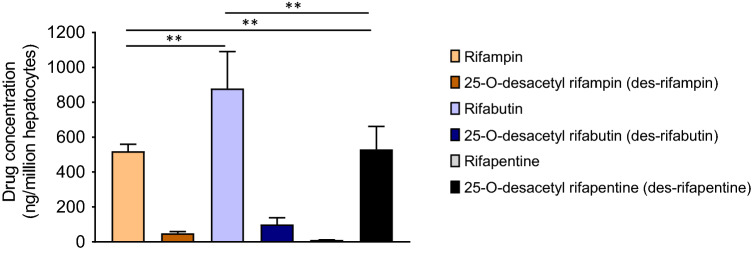
Bioavailability of parent rifampin, rifabutin, and rifapentine drugs and their des-metabolites in primary human hepatocytes (PHHs). PHHs derived from healthy donors were independently treated with rifamycin antibiotics (10 µM) for 72 h. Intracellular concentration of parent to metabolites (Cp/Cm) were quantified using liquid chromatography tandem mass spectrometry (LC/MS) analysis and drug concentration per million PHHs are shown.

PHH associated rifamycin intracellular concentrations (geometric mean) at 72 h post equimolar treatment were: rifabutin (879.1 ± 211.7, mean (ng/million cells) ± standard deviation (SD)), rifampin (519.5 ± 39.4) and rifapentine (10.57 ± 1.1) (Fig. 1). Metabolite to parent (Cm/Cp) ratios of rifampin, rifabutin, and rifapentine to 25-O-desacetyl rifampin (des-rifampin), 25-O-desacetyl rifabutin (des-rifabutin), and 25-O-desacetyl rifapentine (des-rifapentine) metabolites were 0.09, 0.10 and 48.93, respectively (Fig. 1). Results were consistent among three lots of PHHs derived from three independent healthy donors who were free of drug, tobacco, alcohol, and medicine usage (demographic details are listed in Table S1). These results show that in PHHs, rifampin and rifabutin have higher intracellular concentrations than rifapentine due to its rapid metabolism to antimicrobially active des-rifapentine.

### Transcriptomic analysis identified integrated gene signatures of rifamycins in PHHs

**Figure 2:**
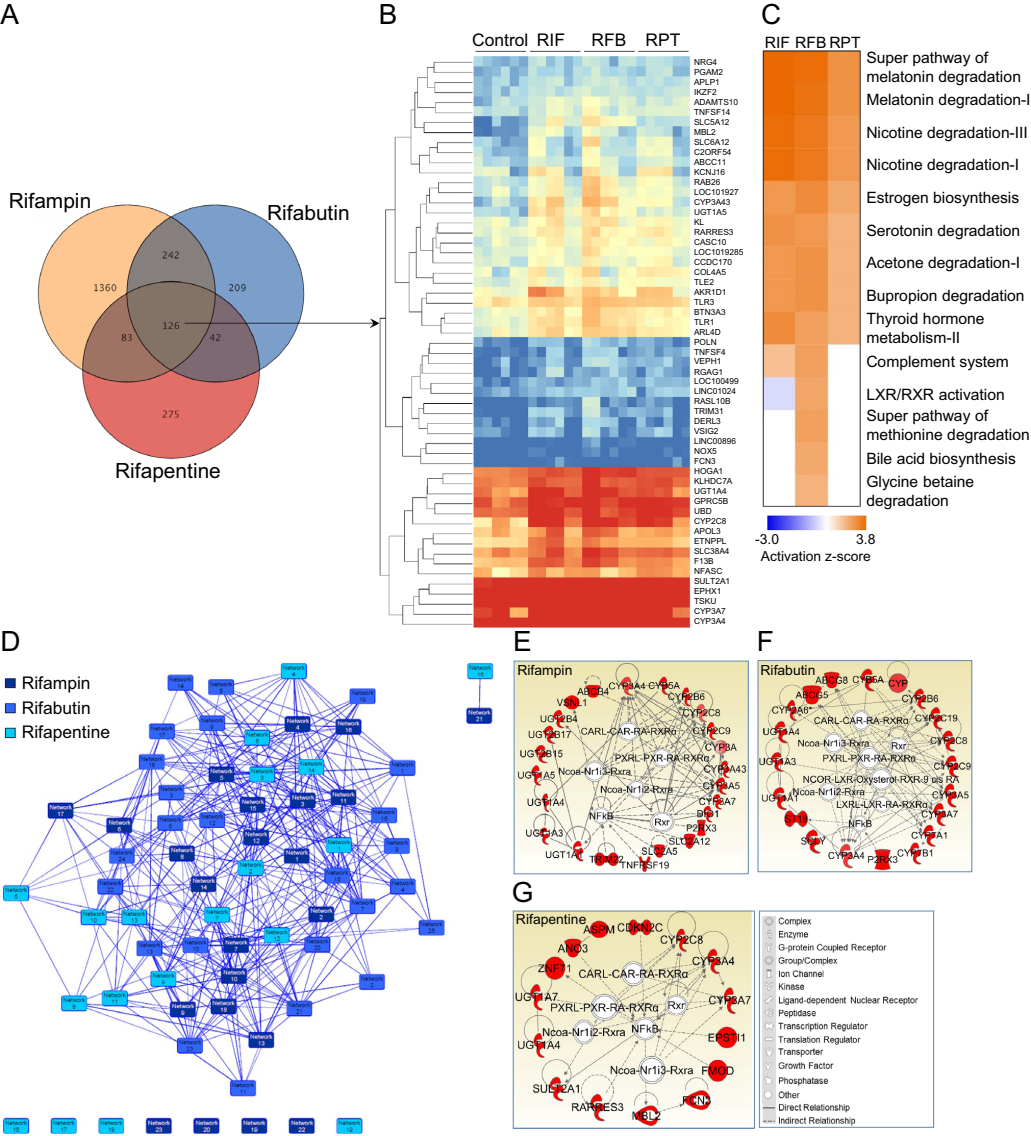
Integrated gene signature of rifamycin antibiotics and pathways induced in PHHs in response to 10 µM of rifampin (RIF), rifabutin (RFB) and rifapentine (RPT) treatment. (**A**) Rifampin, rifabutin, and rifapentine responsive transcripts either uniquely, combinedly, or uniformly regulated among rifamycins in PHHs are shown. (**B**) Heat map shows a total of 126 transcripts that are uniformly regulated among rifamycins, and transcripts were organized based on their level of mRNA expression. (**C**) Biological and metabolic pathways significantly (< 0.01 p value, 0.1 ratio) regulated based on a list of up regulated transcripts in response to rifampin, rifabutin, and rifapentine in PHHs as compared to controls are shown. (**D**) Interactive transcriptional networks of rifampin, rifabutin, and rifapentine responsive genes regulated in PHHs following drug treatments. **(E**) Drug metabolism networks (DMNs) of drug metabolizing enzyme (DME) transcripts specifically induced by rifampin, (**F**) rifabutin, and (**G**) rifapentine built using ingenuity pathway analysis software are shown. Red color indicates the up regulation of a transcript. Transcription factors regulating the expression of drug metabolism genes are shown in the center of the network.

Transcriptomic analysis showed that a number of rifapmin, rifabutin, and rifapentine associated transcripts were significantly (*p* < 0.05) altered (> 1.5 fold change [FC]) 619 (1.53%), 1811 (4.47%), and 526 transcripts (1.3%), respectively in PHHs as compared to vehicle (methanol, 0.0025%) treated controls (Figure S1A, S1B and S1C). Since rifabutin and rifapentine are structural analogues of rifampin, which was originally modified from rifamycin B, we have predicted expression of a unique set of integrated transcripts with similar expression patterns in all rifamycins or in pairs (rifampin and rifabutin, rifabutin and rifapentine, and rifampin and rifapentine). We performed a venn diagram analysis and identified 126 transcripts (0.31%) that were significantly regulated by all rifamycin drugs and constitute an integrated gene signature (Fig. 2A and B). A total of 368 transcripts in rifampin (59.45%) and rifabutin (20.32%), 209 in rifabutin (11.54%) and rifapentine (39.73%), and 168 in rifampin (27.14%) and rifapentine (31.93%) were altered at the transcriptional level in their combinatorial responses in PHHs (Fig. 2B). Interestingly, a total of 20.3% of total rifampin, 6.9% of rifabutin, and 23.9% of rifapentine regulated differentially expressed genes were contributing to the integrated rifamycin gene signature (Fig. 2B). These results showed that < 4.5% of whole transcriptomic changes overlap among the rifamycins and that the responsive transcriptomes contain a large number of transcripts that are characteristically regulated by each rifamycin in PHHs.

### DME profiles in rifamycin responsive PHHs predict lower drug interaction potential of rifapentine

Rifamycin responsive PHH transcriptomes consisted of distinct sets of DMEs. A total of 19 cytochrome P450 genes (CYPs) were induced by rifamycins, and *CYP2A1* was the only down regulated CYP transcript post rifabutin treatment (Table 1). In this analysis, four CYPs, *CYP2C8*, *3A4*, *3A7,* and *3A43*, were integral to all rifamycin responses. *CYP3A4*, a key DME known to regulate the metabolism of a large number of drugs, followed a FC pattern, rifapentine (6.8) < rifampin (16.7) < rifabutin (25.5) (Table 1). Among UDP-glucose-glycoprotein glucosyltransferases (UGTs), 1A4 and 1A5 were induced by rifamycins, *UGT1A1* and *1A3* by rifampin and rifabutin, while others were unique to individual rifampin and rifapentine treatment (Table 1). Among non-CYP classes of genes that regulate drug metabolism, rifamycin induced ATP binding cassette protein (*ABCC*) *11*, apolipoprotein (*APOL*) *3*; rifampin and rifabutin induced ATP binding cassette subfamily B, member 1 (*ABCB1*), *ABCB4*, *ABCC6P1, ABCC7,* and all others were unique to rifamycins (Table S2). Among 56 solute carrier family proteins (*SLCs*), expression of *SLC6A12* and *38A4* were induced, and *42A3* was down regulated by all rifamycins (Table S2). These data showed that rifamycins induced a large number of metabolism-associated genes, but only 6.28% (12 out of 191) of transcripts were integral to all rifamycins, while others were unique to each of the rifamycin drug responses. Expression profiles of *CYP3A4* and other DME transcripts described above showed that rifapentine may have lower DDI potential among rifamycins. Table 1 summarizes the FC of associated DME genes in response to individual rifamycin drug treatment.

**Table 1:** Drug metabolizing enzymes (DMEs) significantly (> 1.5 fold change and < 0.05p value) regulated in response to rifampin (RIF), rifabutin (RFB) and rifapentine (RPT) at 10 μM in primary human hepatocytes (PHHs) as compared to vehicle (methanol, 0.0025%) treated controls. NCBI gene IDs are shown under the column ID#.

Gene	RIF	RFB	RPT	ID#	Gene	RIF	RFB	RPT	ID#	Gene	RIF	RFB	RPT	ID#	Gene	RIF	RFB	RPT	ID#
***Drug metabolizing enzymes (DMEs) ***																			
**I. Oxidative Phase I enzymes**					*B. Monoamine oxidases (MAOs)*					*H. Glutathione peroxidases (GPXs)*					*N. Alkaline phosphatases (ALPs)*				
*A. Cytochrome P 450 enzymes (CYPs) *					MAOB		1.7		4,129	GPX2	2.1			2,877	ALPI	15.6			248
CYP2A1		− 1.7		1543	*C. Aldehyde dehydrogenases (ALDHs)*					*I*.* Carboxypeptidases*					ALPL		2.4		249
CYP2A6	3.5	5.2		1548	ALDH1A2			2.0	8,854	SCPEP1		1.8	1.5	59,342	*O. N-acetyltransferases (NATs)*				
CYP2A7	2.6	5.0		1549	ALDH1L1		4.7	3.1	10,840	CPA4		− 3.2		51,200	NAA15		− 1.5		80,155
CYP2A13	4.2	5.9		1553	ALDH3A1		1.7		218	CPO		1.7		130,749	SAT2		1.5		112,483
CYP2B6	6.1	3.7		1555	ALDH5A1		1.5		7,915	CPVL		1.6	1.9	54,504	*P. Amino acid conjugating enzymes*				
CYP2B7P	3.6	3.5	5.1	1556	ALDH6A1		1.8		4,329	*J. Endopeptidases*					ACSL4		− 2.2		2,182
CYP2C8	13.5	18.1		1558	ALDH8A1		2.4		64,577	PHEX		1.5		5,251	ACSL6		1.8		23,305
CYP2C9	2.7	4.3		1559	**II. Reductive phase I DMEs**					**IV. Conjugative phase II DMEs**					ACSM1		3.1		116,285
CYP2C19		1.8		1557	*D. Aldo–keto reductases (AKRs)*					*K. UDP glucuronosyl transferases (UGTs)*					ACSM2A	1.8	3.1		123,876
CYP2S1			2.0	29,785	AKR1B1	1.9		2.7	231	UGT1A1	2.5	2.9		54,658	ACSM2B		2.8		348,158
CYP2W1		2.1		54,905	AKR1D1	4.5	2.2	2.2	6,718	UGT1A3	1.8	2.0		54,659	ACSM3		2.4		6,296
CYP3A4	16.7	25.5	6.8	1576	AKR1A1		1.5		10,327	UGT1A4	3.6	3.5	1.5	54,657	ACSS1		2.1		84,532
CYP3A5	2.6	3.7		1577	AKR1B10		− 3.7		57,016	UGT1A5	6.2	5.5	2.1	54,579	GLYAT		3.1		10,249
CYP3A7	6.2	7.3	3.3	1551	AKR1B15		− 2.5	− 3.0	441,282	UGT1A8			1.8	54,576	LPCAT4		− 1.6		254,531
CYP3A43	5.8	9.1	3.1	64,816	AKR1C6P		3.1		389,932	UGT1A9			1.6	54,600	LRAT		1.9		9,227
CYP4V2		1.7		285,440	AKR7A2P1			1.7	246,182	UGT1A10			1.5	54,575	*Q. Methyl Transferases (MTs)*				
CYP7A1		9.4		1581	*E. Quinone reductases*					UGT2B4	1.6			7,363	AS3MT		1.5		57,412
CYP7B1		1.6		9,420	NQO1		− 2.5		1728	UGT2B15	2.2			7,366	BHMT		3.4	1.6	635
CYP11A1		1.8	1.7	1583	**III. Hydrolytic Phase I DMEs**					UGT2B17	2.3			7,367	DOT1L		− 1.5		84,444
CYP21A1P	1.5			1,590	*F. Epoxide hydrolases (EPHXs)*					*L. Sulfotransferases (SULTs)*									
Other Cytochrome transcripts					EPHX1	2.7	3.2	1.7	2052	SULT2A1	2.5	3.8	2.1	6,822	HNMT		1.6		3,176
CYBA			2.2	1535	*G. Aminopeptidases*					SULT4A1			2.0	25,830	METTL7A		1.5		25,840
CYB5A	2.2	1.6		1528	ENPEP		1.9		2028	*M. Glutathione S-transferases (GSTs)*					METTL7B		− 1.5		196,410
CYB5D2		1.5		124,936	ERAP1		1.7		51,752	GSTM2	1.7	2.0		2,946	NNMT		− 2.3		4,837
CYB5RL		1.7		606,495	ASPRV1	1.8			151,516	GSTT2B			1.7	653,689	TRMT10A		− 1.6		93,587
CYBRD1			1.9	79,901	MASP1	1.5	2.5		5,648										

### Lower interaction potential of rifapentine with therapeutics used to treat HIV, cancer and other co-morbidities in TB patients

One of the major objectives of our study was to identify potential interactions of rifamycins with drugs used to treat common comorbidities in TB patients. In this process, DME expression profiles from the list of rifamycin-RGs were an important resource to predict clinical DDIs. The most important qualitative features of NGS are (i) sequence accuracy and (ii) gene features, including, coding (exon), non-coding (intron), intergenic, and untranslated regions. RNA profiles of our study samples showed near to 100% perfect index reads and maintained similar exon and intron sequences among replicates of rifampin, rifabutin, rifapentine treated and control (methanol and untreated) samples (Figures S2 and S3), which confirmed their quality and improved confidence in our data. With the disease and function annotation analysis tool in ingenuity pathway analysis software, we predicted the interaction of rifamyins with 58 FDA-approved drugs. Among these drugs, 17 were used to treat HIV, 10 to treat cancer, one to treat malaria and two to treat fungal infections (Table 2). Among anti-HIV drugs, protease inhibitors, including, atazanavir, darunavir, fosamprenavir, lopinavir, saquinavir, tipranavir, and the non-nucleoside reverse transcriptase inhibitor, rilpivirine, and pharmacokinetic enhancers, cobicistat and ritonavir, are all readily metabolized by hepatic *CYP3A4* (Table 2). Based on CYP3A4 regulation, we have identified a comparative DDI pattern of rifapentine < rifampin < rifabutin (Table 2). Rifampin and rifabutin induced UGT1A1, which is involved in raltegravir and abacavir metabolism (Table 2) with a comparative DDI pattern, rifapentine < rifampin < rifabutin. Multiple DMEs are involved in the major metabolism of many antiretroviral drugs, including dolutegravir, elvitegravir, efavirenz, etravirine, and nevirapine (Table 2), based on deducibility, rifapentine may have lower drug interaction potential as compared to rifampin and rifabutin when used in combination with these antiretrovirals. Disease and function annotation analysis predicted interaction of rifamycins with other drugs used to treat multiple diseases in TB patients (Table 3). These data summarize the DDI potential of rifamycins and indicate a lower interaction potential of rifapentine among rifamycins.

**Table 2:** Predicted drug-drug interaction (DDI) potential of rifamycin antibiotics with therapeutics used to treat HIV, fungal, malarial parasitic infections and cancer in tuberculosis patients.

Interacting drugs	Drug metabolizing enzymes induced during treatment			DDI pattern
	Rifampin (RIF)	Rifabutin (RFB)	Rifapentine (RPT)	
**Antiretroviral Drugs (ARVs)**				
ARV Category I: Integrase Strand Transfer Inhibitors (INSTIs)				
Dolutegravir	CYP3A4, UGT1A1	CYP3A4, UGT1A1	CYP3A4	RPT < RIF < RFB
Bictegravir	CYP3A4, UGT1A1	CYP3A4, UGT1A1	CYP3A4	RPT < RIF < RFB
Elvitegravir	CYP3A4, UGT1A1, UGT1A3	CYP3A4, UGT1A1, UGT1A3	CYP3A4	RPT < RIF < RFB
Raltegravir	UGT1A1	UGT1A1		RPT < RIF < RFB
ARV Category II: Non-nucleoside Reverse Transcriptase Inhibitors (NNRTIs)				
Efavirenz	CYP2A6, 2B6	CYP2A6, 2B6, 2C19		RPT < RIF < RFB
Etravirine	CYP3A4, 2C9	CYP3A4, 2C9, 2C19	CYP3A4	RPT < RIF < RFB
Nevirapine	CYP3A4, 2B6	CYP3A4, 2B6	CYP3A4	RPT < RIF/RFB
Rilpivirine	CYP3A4	CYP3A4	CYP3A4	RPT < RIF < RFB
ARV Category III: Nucleoside Reverse Transcriptase Inhibitors (NRTIs)				
Abacavir	UGT1A1	UGT1A1		RPT < RIF < RFB
ARV Category IV: Protease Inhibitors (PIs)				
Atazanavir	CYP3A4	CYP3A4	CYP3A4	RPT < RIF < RFB
Darunavir	CYP3A4	CYP3A4	CYP3A4	RPT < RIF < RFB
Fosamprinavir	CYP3A4	CYP3A4	CYP3A4	RPT < RIF < RFB
Lopinavir	CYP3A4	CYP3A4	CYP3A4	RPT < RIF < RFB
Saquinavir	CYP3A4	CYP3A4	CYP3A4	RPT < RIF < RFB
Tipranavir	CYP3A4	CYP3A4	CYP3A4	RPT < RIF < RFB
ARV Category IV: Entry Inhibitors (EIs)				
Maraviroc	CYP3A4	CYP3A4	CYP3A4	RPT < RIF < RFB
ARV Category V: Pharmacokinetic enhancers (PK boosters)				
Cobicistat	CYP3A4	CYP3A4	CYP3A4	RPT < RIF < RFB
Ritonavir	CYP3A4	CYP3A4	CYP3A4	RPT < RIF < RFB
**Antifungals**				
Terbinafine	CYP2B6, 2C8, 2C9, 3A4, 3A5	CYP2B6, 2C19, 2C8, 2C9, 3A4, 3A5	CYP2C8, 3A4	RPT < RIF < RFB
Voriconazole	CYP2C9,CYP3A5	CYP2C9,CYP2C19,CYP3A5	-	RPT < RIF < RFB
**Antimalarials**				
Quinine	CYP3A4	CYP3A4	CYP3A4	RPT < RIF < RFB
**Anticancer Drugs**				
Beta-estradiol	CYP3A4, 3A5, 3A7	CYP3A4, 3A5, 3A7	CYP3A4, 3A7	RPT < RIF < RFB
Cyclophosphamide	CYP2A6, 2B6, 2C8, 2C9, 3A4	CYP2A6, 2B6, 2C19, 2C8, 2C9, 3A4	CYP2C8, 3A4	RPT < RIF & RFB
Docetaxel	CYP3A4, 3A5	CYP3A4, 3A5	CYP3A4	RPT < RIF < RFB
Etoposide	CYP2C9, 3A4, 3A5	CYP2C9, 3A4, 3A5	CYP3A4	RPT < RIF < RFB
Ifosfamide	CYP2A6, 2B6, 2C8, 2C9, 3A4	CYP2A6, 2B6, 2C19, 2C8, 2C9, 3A4	CYP2C8, 3A4	RPT < RIF & RFB
Omeprazole	CYP2A6, 2C9, 3A4	CYP2A6, 2C19, 2C9, 3A4	CYP3A4	RPT < RIF < RFB
Paclitaxel	CYP2B6, 2C8, 3A4, 3A5	CYP2B6, 2C8, 3A4, 3A5	CYP2C8, 3A4	RPT < RIF & RFB
Tamoxifen	CYB5A, CYP2B6, 2C9, 3A4, 3A5; UGT2B15	CYP5A, 2B6, 2C19, 2C9, 3A4, 3A5	CYP3A4	RPT < RIF & RFB
Thalidomide	CYP2C9, 3A5	CYP3A5		RPT < RIF < RFB
Tretinoin	CYP2B6, 2C8, 2C9, 3A4, 3A5, 3A7	CYP2B6, 2C8, 2C9, 3A4, 3A5, 3A7, RDH16, ALDH8A1	CYP2C8, 2S1, 3A4,3A7	ND

Abbreviations: CYP, cytochrome P450; UGT, UDP glucuronosyl transferase; RDH16, Retinol dehydrogenase 16 and ALDH8A1, aldehyde dehydrogenase 8 family member A1 and ND, not determined.

**Table 3:** Predicted Drug-Drug Interactions (DDIs) of rifamycins with therapeutics used to treat various illnesses in TB patients.

Interacting drugs	Drug metabolizing enzymes induced during treatment			Predicted DDI pattern
	Rifampin (RIF)	Rifabutin (RFB)	Rifapentine (RPT)	
**Anti-inflammatory drugs**				
Indomethacin	**-**	CYP2C19, CYP2C9	**-**	RPT < RIF < RFB
Colchicine	CYP3A4	CYP3A4	CYP3A4	RPT < RIF < RFB
**Anti-diabetic drugs**				
Tolbutamide	CYP2C8, 2C9, 3A5	CYP2C8, 2C9, 2C19, 3A5	CYP2C8	RPT < RIF < RFB
**Immunosuppressive drugs**				
Sirolimus	CYP3A4	CYP3A4	CYP3A4	RPT < RIF < RFB
**Antiarrhythmic drugs**				
Amiodarone	CYP2C8, 3A4, 3A5	CYP2C8, 2C19, 3A4, 3A5	CYP2C8, 3A4	RPT < RIF < RFB
Lidocaine	CYP3A4	CYP3A4	CYP3A4	RPT < RIF < RFB
**Antihypertensive drugs**				
Verapamil	CYP2C8, 3A4, 3A5	CYP2C8, 3A4, 3A5	CYP2C8, 3A4	RPT < RIF < RFB
**Antidepressant drugs**				
Bupropion	CYP2B6, 3A4	CYP2B6, 3A4	CYP3A4	RPT < RIF < RFB
Imipramine	CYP2B6, 3A4, UGT1A4	CYP2B6, 2C19, 3A4, UGT1A4	CYP3A4, UGT1A4	RPT < RIF & RFB
Morphine	CYP2C8, 3A4	CYP2C8, 3A4	CYP2C8, 3A4	RPT < RIF < RFB
**Anticonvulsant drugs**				
Carbamazepine	CYP2C8, 3A4, 3A5, 3A7	CYP2C8, 3A4, 3A5, 3A7	CYP2C8, 3A4, 3A7	RPT < RIF < RFB
**Antipsychotic drugs**				
Haloperidol	CYP3A4, 3A5	CYP3A4	CYP3A4	RPT < RIF < RFB
**Antisedative drugs**				
Midazolam	CYP2B6, 3A4, 3A5	CYP2B6, 3A4, 3A5	CYP3A4	RPT < RIF & RFB
Triazolam	CYP3A4, 3A5	CYP3A4, 3A5	CYP3A4	RPT < RIF < RFB
Alprazolam	CYP3A4, 3A5	CYP3A4, 3A5	CYP3A4	RPT < RIF < RFB
Diazepam	CYP2B6, 2C9, 3A4, 3A5	CYP2B6, 2C19, 2C9, 3A4, 3A5	CYP3A4	RPT < RIF & RFB
**Vasodilators**				
Sildenafil	CYP2C9, 3A4	CYP2C9,CYP3A4	CYP3A4	RPT < RIF < RFB
**Analgesics**				
Methadone	CYP2B6, 2C8, 2C9, 3A4	CYP2B6, 2C8, 2C9, 2C19, 3A4	CYP2C8, 3A4	RPT < RIF & RFB
Ibuprofen	CYP2C8, 2C9	CYP2C8, 2C9, 2C19	CYP2C8	RPT < RIF < RFB
**Sedatives**				
Midazolam	CYP2B6, 3A4, 3A5	CYP2B6, 3A4, 3A5	CYP3A4	RPT < RIF/RFB
Triazolam	CYP3A4, 3A5	CYP3A4, 3A5	CYP3A4	RPT < RIF < RFB
Alprazolam	CYP3A4, 3A5	CYP3A4, 3A5	CYP3A4	RPT < RIF < RFB
Diazepam	CYP2B6, 2C9, 3A4, 3A5	CYP2B6, 2C19, 2C9, 3A4, 3A5	CYP3A4	RPT < RIF & RFB
**Orexogenic drugs**				
Dronabinol	CYP3A4	CYP3A4	CYP3A4	RPT < RIF < RFB
**Adrenocortical insufficiency treating drugs**				
Hydrocortisone	CYP3A4, 3A5, FOXA1	CYP3A4	CYP3A4	RPT < RIF < RFB
**Lipid-lowering drugs**				
Fluvastatin	CYP2C8, 2C9, 3A4	CYP2C8, 2C9, 3A4	CYP2C8, 3A4	RPT < RIF < RFB
Simvastatin	CYP2C9, 3A4	CYP2C9, 3A4	CYP3A4	RPT < RIF < RFB
Pravastatin	CYP3A4	CYP3A4	CYP3A4	RPT < RIF < RFB
**Hormonal drugs**				
Testosterone	CYP3A4, 3A5	CYP3A4	CYP3A4	RPT < RIF < RFB
**Generalized anxiety disorder (GAD) treated drugs**				
Escitalopram	CYP3A4	CYP2C19, 3A4	CYP3A4	RPT < RIF < RFB
**Smoking cessation aiding drugs**				
Nicotine	CYP2A6, 2B6	CYP2A6, 2B6		RPT < RIF & RFB

CYP, cytochrome P450 and UGT, *UDP* glucuronosyl transferase.

### Rifamycins are strongest but selective stimulants of drug metabolism pathways in PHHs

A total of 327, 441, and 297 pathways were induced in response to rifampin, rifabutin, and rifapentine treatment in PHHs respectively. Of these, activated biological pathways, 9 (2.75%), 13 (2.94%), and 7 (2.35%) were found to be significant at *p* = 0.01, 0.1 ratio and 2.0 Z score. In this, seven pathways were stimulated by all rifamycins (Fig. 2C). Interestingly, several of the activated drug metabolizing pathways involved *CYP2C8*, *3A4*, *3A7*, *UGT1A4*, *1A5*, and sulfotransferase family 2A member 1 (*SULT2A1*) DMEs as key modulators (Table S3 and S4), which are also part of the integrated rifamycin transcriptional signature (Fig. 2B and Table S3). While rifampin-RGs independently regulated the serotonin degradation pathway, rifabutin-RGs stimulated several metabolic pathways, such as degradation of glycine, betaine, and methionine and bile acids biosynthesis as shown in Table S4. Even in down regulated pathways, rifabutin inhibited 9 cell signaling and 2 metabolic pathways, including geranyl diphosphate and cholesterol biosynthesis (Figure S4 and Table S5). Rifampin and rifapentine did not significantly inhibit any pathways and showed their distinction from rifabutin regulatory transcriptional pathways. These results showed that rifamycins strongly stimulate drug metabolic pathways in PHHs involving *CYP2C8*, *3A4*, *3A7*, *UGT1A4*, *1A5,* and *SULT2A1* as key regulators.

### Interactive transcriptional drug metabolism networks predict the role of *NCOA*, *NF-KB, NR1I2*, *NR1I3 and RXRα* transcription factors in regulation of DMEs in rifamycin responsive PHHs

A tightly regulated gene network is dedicated to perform a specific function in drug responsive cellular transcriptomes. From a network analysis of rifampin, rifabutin, and rifapentine regulatory DME profiles, we found 23 rifampin, 25 rifabutin, and 19 rifapentine gene networks are operated in PHHs following their independent treatments (Fig. 2D). In rifamycin responsive gene networks, 1 through 18 rifampin, all of rifabutin, and 1 through 14 rifapentine regulated networks were dedicated to cell signaling and drug metabolism functions. Among transcriptomic networks, rifampin, rifabutin, and rifapentine responsive 2nd, 6th, and 10th networks were specifically associated with drug metabolism.

These drug metabolism networks (DMNs) involve ≤ 24 DMEs; *CYP2C8*, *3A4*, and *3A7*; *UGT1A4* and *1A5* were integral to these networks (Fig. 2E–G). No common transcripts were found in either rifabutin and rifapentine or rifampin and rifapentine combinedly regulated networks. However, both rifampin and rifabutin regulated DMEs, including, *CYP2A6*, *2B6*, *2C9*, *3A5*, *UGT1A1*, *1A5*, and *P2RX3* in DMNs (Fig. 2E and F). These data demonstrate the distinction of rifapentine from rifampin and rifabutin functional responses in PHHs and also suggest rifapentine may have lower DDI potential with drugs metabolized by these transcripts. Along with more than one rifamycin antibiotic regulated genes, drug metabolizing networks had a set of genes that were unique to each of the rifamycin drugs in their DMNs (Fig. 2E–G). DMNs have provided valuable data on rifamycin regulated *NCOA*- *NR1i2*/*NR1i3*-*RXRα*; *RXR*; *NF-KB*; *NR1I2L*-*NR1I2*-RA-*RXRα* transcription factor (TF) axes controlling the expression of downstream DME targets involved in drug metabolism (Fig. 2E–G). Along with above results, rifampin and rifabutin together controlled DMN through downstream *NR1I3L*-*NR1I3*-RA-*RXRα* TF axis, and rifabutin regulated DMN was specifically controlled by *NCOR*-*LXR*-Oxysterol-*RXR*-9-Cis RA TF axis (Fig. 2E, F). These data conclude that more than 14 rifamycin responsive transcriptional networks are operated by key TFs, *NR1I2*, *NR1I3* (*NR1I3*), *NCOA*, *RXRα* and *NF-KB*, which control DME expression in DMNs of rifamycin responsive PHH transcriptomes.

### Upstream regulator analysis of rifamycin responsive genes predict *FOXA3*, *HNF4α*, *NR1I2*, *NR1I3*, *NR3C1,* and *RXRα* as key regulators of metabolic programs in PHHs

Further validation of TF axes involving *NCOA, NF-KB, NR1I2*, *NR1I3* and *RXRα* was performed using the upstream regulator analysis (URA) tool in IPA. In this analysis, we allocated rifamycin-RGs containing DMEs and drug transporters as target genes of TFs, which may control their mRNA expression. URA predicted *FOXA3*, *HNF4α*, *NR1I2*, *NR1I3*, *NR3C1*, and *RXRα* TFs are potentially involved (< 0.05 *p* and > 2.0 z score), and each regulated more than 10 DMEs and drug transporter targets from the list of rifamycin-RGs (Table 4 and Table S6). Based on z (> 1.0) and p value (< 0.01) based measures, all rifamycin drugs significantly activated rifamycin-RGs controlled by *NR1I3*, whereas both rifampin and rifabutin-RGs were targets of *HNF4α*, *NR1I2*, *NR3C1*, and *RXRα* and showed higher specificity to control rifabutin-RG targets (Table 4 and Table S6). *NR1I3* heterodimerizes with *RXRα* and regulates a distinct set of metabolic genes in hepatocytes. In our dataset, we found 20 rifamycin-RGs as *NR1I3* targets and 1 (5%) was induced by rifampin, 5 (25%) by rifabutin, 8 (40%) by both rifampin and rifabutin, 1 (5%) by both rifabutin and rifapentine, and 5 (25%) by all rifamycins (Table 4 and Table S6). In the liver, *HNF4α* and *NR1I2* are specifically expressed to higher levels. In our dataset, *HNF4α* was predicted to regulate 145 rifamycin-RG targets, and in this analysis 10.3% rifampin (15 genes), 56.5% rifabutin (82 genes), and 9.6% rifapentine (14 genes) were specific to individual rifamycins, and 13.1% to rifampin and rifabutin (19 genes), 2.7% to rifabutin and rifapentine (4 genes), and 7.5% to all rifamycins (11 genes) (Table S6). Notably, the *NR1I2* receptor bound to rifampin, rifabutin, and rifapentine and induce expression of its target genes. A total of 22 rifamycin-RGs were identified as *NR1I2* targets in our dataset, including 10 regulated by rifabutin (45.4%), 7 by both rifampin & rifabutin (31.8%), 1 by both rifabutin and rifapentine (4.5%), and 5 by all rifamycins (22.7%) (Table S6). *NR1I2* heterodimerizes with *RXRα* to regulate expression of a unique set of metabolic genes. In a total of 34 *RXRα*’s rifamycin-RG targets, 5 were regulated by rifampin (14.7%), 12 by rifabutin (35.2%), 4 by rifapentine (11.7%), 10 by both rifampin and rifabutin (29.4%), and 3 (8.8%) by all rifamycins (Table S6). Induction of *NR3C1* TF was predicted based on it’s association with 55 targets in URA. In this analysis, 8 genes (14.5%) were regulated by rifampin, 24 (43.6%) by rifabutin, 7 (12.7%) by rifapentine, 7 (12.7%) by both rifampin and rifabutin, 7 (12.7%) by all rifamycins, and 1 (1.8%) by rifampin & rifapentine (Table S6). *CYP2C8*, *3A4*, and *3A8* were key DMEs that metabolized a wider array of drugs that are controlled by multiple TFs, including, *FOXA3*, *HNF4α*, *NR1I2*, *NR1I3*, and *NR3C1* (Table S6). In summary, URA results showed that *HNF4α*, *NR1I2*, *NR1I3*, *FOXA3*, *NR3C1*, and *RXRα* are key TFs controlling expression of key metabolic genes, including *CYP3A4*.

**Table 4:** Transcription factors (TFs) predicted to control the expression of target gens induced during rifampin (RIF), rifabutin (RFB) and rifapentine (RPT) treatment in hepatocytes that further regulate drug metabolism networks were identified by upstream regulator analysis tool in IPA software.

TFs	*p* Value	Z Score	Status	*p* Value	Z Score	Status	*p* Value	Z Score	Status
FOXA3	1.21E−05	2.216	Activated	3.78E−06	1.678	–	6.87E−03	−	–
HNF4α	6.01E−04	2.242	Activated	5.42E−08	4.086	Activated	1.56E−02	0.963	–
NR1I2	2.35E−07	3.542	Activated	5.00E−10	2.471	Activated	5.33E−03	1.768	–
NR1I3	9.63E−12	3.678	Activated	1.88E−10	3.396	Activated	6.41E−04	2.39	Active
NR3C1	2.07E−05	1.982	–	8.21E−04	2.376	Activated	4.06E−03	0.348	–
RXRα	3.21E−08	1.451	–	4.53E−06	2.415	Activated	6.29E−03	–	–

## Discussion

Previous in vitro approaches to examine the DDI potential of rifamycins have either used real time polymerase chain reaction (RT-PCR) or microarray technologies to quantify changes in the gene expression of DMEs^16,20–24^. The results from these studies have provided some evidence on DME expression pattern in PHHs, and may differ from a highly sensitive NGS approach^25^. Moreover, existing evidence on rifabutin and rifapentine DDI potential is limited. We overcame several of these challenges with the use of (1) PHHs from healthy donors free of drug, tobacco, alcohol, and medicine usage (Table S1); (2) the use of NGS technology to precisely quantitate changes in gene expression; and (3) comparison of rifampin, rifabutin, and rifapentine on a similar platform using equimolar drug treatment concentrations (10 μM) to demonstrate comparative DME gene expression.

Deciphering transcriptome based metabolic responses to a drug in PHHs is an important “in vitro” strategy to identify potential DDIs. In transcriptomic responses, changes in DMEs and drug transporter patterns further influence metabolic pathways, interactive transcriptional networks, and upstream regulators, all of which provide significant information on potential drug interactions and molecular events underlying DME profiles and their associated DDIs. In our experiments, the initial quantitation of intracellular concentrations of rifampin, rifabutin, and rifapentine and their active desacetyl-metabolites in PHHs has demonstrated the relative metabolic rates of rifamycins in PHHs. We found that the metabolic rates followed a pattern of rifapentine > rifabutin ≥ rifampin, which formed an important basis for downstream transcriptomic studies.

Both rifapentine and 25-desacetyl-rifapentine have antimicrobial properties, which may contribute to its prolonged antimicrobial activity^26^, and its therapeutic effectiveness has been observed in recent clinical studies^27-29^. While rifamycins share a structure–activity relationship among themselves, there are some considerable differences observed in their antimicrobial and other pharmacologic properties: (1) rifabutin has enhanced antimicrobial activity against *Mycobacterium avium* and its potency was found to be similar to rifampin against *M. tuberculosis*^30^; (2) the half-life (t1/2) of rifabutin (32–67 h) > rifapentine (14–18 h) > rifampin (2–5 h) in the serum of treated TB patients^31,32^ with the relative protein binding ability of rifapentine (97.7%) > rifampin (≤ 88%) > rifabutin (85%)^33–35^; (3) maximal concentrations (C_max_) of rifamycins observed in the serum of rifamycin treated TB patients follow a rifapentine (≤ 30 mg/L, 600 mg single daily dose) ≥ rifampin (≤ 20 mg/L, 600 mg) > rifabutin (≤ 0.6 mg/L, 300 mg) pattern with the clinically used doses^32^ and (4) all rifamycins undergo deacetylation and form ‘deacetyl’ derivatives, but rifampin and rifapentine uniquely undergo hydrolysis and form ‘formyl’ derivatives, whereas rifabutin undergoes hydroxylation and forms ‘hydroxyl’ derivatives. A comparison of transcripts induced in response to each of the rifamycins in PHHs has not been identified to date. This study showed that 126 transcripts were integrally regulated by all rifamycins, and rifampin, rifabutin, and rifapentine contributed 1.53%, 4.47%, and 1.3% of total transcripts in PHHs, respectively (Fig. 2A). About 1.22%, 4.15%, and 0.99% of total transcripts were uniquely regulated in response to rifampin, rifabutin, and rifapentine responses in PHHs, respectively. Relatedly, several metabolic pathways were significantly induced by all rifamycins.

In drug metabolism, *CYP3A4* is a key common metabolic enzyme involved in clearance of > 80% of currently used therapeutics^36^. This is the first RNA sequence-based report showing the *CYP3A4* induction pattern among rifamycins as rifapentine < rifampin < rifabutin (Table 1). Previously, Dooley et al.^37^ showed that when midazolam was combined with rifapentine, midazolam had faster clearance rate as compared to midazolam combined with rifampin, potentially due to higher *CYP3A4* activity. In contrast, Li et al., showed that comparative induction of *CYP3A4* acvtivity among rifamycins was rifampin > rifapentine > rifabutin based on 6ß- hydroxylation of testosterone as an indirect measure of *CYP3A4* activity^21^. Later, Williamson et al. showed a different pattern of rifampin > rifabutin > rifapentine based on in vitro real-time PCR experiments. Our data are consistent with the report from Williamson et al.^23^, which showed that rifapentine was identified as the weakest *CYP3A4* inducer among rifamycins on an equimolar basis. Whether differences in the level of absorption of rifamycin antibiotics in the gut of TB infected persons contribute to the different *CYP3A4* induction pattern of rifamycin drugs in “in vivo” studies is unclear.

Consistent with earlier reports, metabolism of melatonin^38,39^ and bupropion^40^ were increased, along with acetone and nicotine metabolism, based on rifampin mediated DME expression (Table S3). We found rifapentine to be the weakest inducer of *CYP2C8*, *3A4*, *3A7*, and *UGT1A4* and *1A5* as compared to rifampin and rifabutin at similar concentrations (10 µM). A previous clinical study by Burman et al.^32^ showed that the use of a *CYP3A4* inhibitor enhanced the bioavailability of rifabutin, whereas the inhibitor could not boost rifampin and rifapentine bioavailability. Based on our study results, rifabutin was the highest inducer of CYP3A4 and is a major pathway involved in its clearance and thereby it’s inhibition may have increased it’s bioavailability.

Healthy volunteer studies of the antiretroviral drug raltegravir in combination with rifapentine, rifampin or rifabutin are consistent with our findings. Combination of raltegravir (400 mg twice daily) with rifapentine (600 mg once daily) showed an decrease in raltegravir’s AUC 0–12 h by 5% as compared to raltegravir alone^41^. In contrast, both rifampin (600 mg single daily dose) and rifabutin (300 mg single daily dose) reduced raltegravir AUC 0–12 h by 41% and 19% respectively^42,43^. However, regimens used to either treat or prevent TB often utilize multiple drugs in combination, such as rifampin or rifapentine in combination with isoniazid, making interpretation of possible drug-drug interactions difficult. More so, differences exist in the adminsitration of rifamycins between regimens, including various doses and dosing schedules. Collectively, these differences hinder the ability to directly compare regimens and their effects on DMEs.

Unlike rifampin and rifabutin, rifapentine may not influence a large number of metabolic pathways as it showed a higher specificity among rifamycins. Rifapentine is combined with isoniazid for treatment of LTBI and a one month regimen was shown non-inferior to nine months of isoniazid or four months of rifampin or three months of rifapentine and isoniazid therapy^44^. In the case of DME inducing ability, all rifamycins induced *UGT1A4*, *1A5*, and *SULT2A1*. In addition, rifampin and rifabutin combinedly induced *UGT1A1* and *1A3*, while rifampin induced *UGT2B4*, *2B17*, and *2B15*, which indicated their additional influence with potential drug metabolizing pathways as compared to rifapentine (Table S3).

The specific metabolic programs influenced by treatment of PHHs with rifamycins were evident through analysis of interactive transcriptional networks. In our analysis, we identified *NF-KB*, *NCOA*, *NR1I2*, *NR1I3* and *RXRα* TFs as key regulators of a complex network of DMEs and drug transporters. Additional evidence from URA showed the involvement of *FOXA3*, *HNF4α*, *NR1I2*, *NR1I3*, *NR3C1* and *RXRα* TFs on regulation of CYPs and other key metabolic genes in PHHs. The role of *FOXA3* and *NR3C1* on controlling the expression of DMEs in PHHs has largely remains unexplored. Though this study has provided a strong evidence on the regulation of above TFs in controlling the expression of DMEs during rifamycin treatment, additional evidence supporting our data may strengthen these findings and fill in the important gaps in DDIs that arise with the use of rifamycins. Though this study has discussed consistency of rifamycin DDI pattern with clinical data on rifamycins and their interactions with raltegravir, additional supportive clinical data may be needed on rifamycin DDIs with various drugs used to treat co-morbidities in TB patients. Future studies may provide additional evidence to show clinical significance of effective therapies designed based on our study to treat co-morbidities in TB patients.

In conclusion, our data has provided evidence on relative changes in DMEs and drug transporter profiles altered in response to rifampin, rifabutin, and rifapentine treatment. Additionally, this study illustrated the lower interaction potential of rifapentine among rifamycins with concomitant medications such as antiretroviral and anticancer drugs. Finally, it confirms the involvement of *FOXA3*, *HNF4α*, *NR1I2*, *NR1I3*, *NR3C1* and *RXRα* TFs in regulation of DMEs including *CYP3A4*.

## Materials and methods

### Primary human hepatocytes (PHHs)

Human plateable induction-qualified PHHs isolated from three drug/tobacco/alcohol free healthy donors were purchased from Life technologies corporation (Chicago, IL) or Lonza (Chicago, IL) (additional details described in Table S1). Reagents required to culture PHHs were purchased from Life technologies corporation, Chicago, IL unless otherwise noted. Cells were thawed at 37 °C for one minute and were transferred into 50 mL of hepatocyte thawing medium (CAT# CM7500) and the tube was centrifuged at 100 g for 10 min. PHHs were seeded at 0.5 million cells per well density in a collagen coated (12–18 h) six well plate (CAT#A1142802). PHHs were then cultured in Williams E medium without phenol red (CAT#A1217601) supplemented with Hepatocyte thawing and plating supplements (CAT#CM3000) and incubated at 37 °C. Medium was replaced with Williams E medium without phenol red supplemented with Hepatocyte maintenance cocktail (CAT#CM4000) and were incubated for 48 h with daily changes of culture medium before drug treatments.

### Drugs, treatments and drug analysis

Rifampin and rifabutin were purchased from US Pharmaceuticals, Rockville, MD and rifapentine was a kind gift of Sanofi-Aventis, Paris, France. PHHs were treated with rifampin, rifabutin and rifapentine at 10 µM concentration for 72 h. with the corresponding untreated (negative) and 0.0025% methanol (vehicle) treated controls. We measured intracellular concentrations (ICC) of parent and des-acetyl-metabolites by liquid chromatography tandem mass spectroscopy (LC–MS/MS) as we have previously described^45–49^.

### RNA Isolation, NGS and differential gene expression analysis

Total RNA, was extracted from rifampin, rifabutin, rifapentine and vehicle treated and untreated PHHs using RNeasy fibrous tissue mini kit (Qiagen, Carol Stream, IL) according to the manufacturer’s protocol and as described previously^51,52^. To quantify copies of transcripts expressed in PHHs in response to rifampin, rifabutin and rifapentine, we performed NGS using the Illumina HiSeq 2500 sequencer available at the University of Nebraska Medical center (UNMC)’s genomics core facility at UNMC, Omaha, NE. Complimentary DNA (cDNA) libraries for rifamycin treated PHH samples were constructed using the TruSeq RNA Library Preparation Kit (Illumina, San Diego, CA). All samples were subjected to 50-cycle, single-read sequencing in the HiSeq2500 and were demultiplexed using Bcl2Fastq v2.17.1.14 (Illumina, San Diego, CA). Short cDNA fragment data were compiled in FASTQ format. Further analysis of FASTQ files were performed in strand NGS software (Agilent technologies, USA). Gene expression levels were calculated using fragments per kilobase of transcript per million mapped reads (FPKM) following normalization. RNA sequencing data files and processed transcript expression are available at NCBI GEO (Accession No# GSE139896). The data obtained from sequence analysis were of high quality (> 98.5% perfect index reads or PIR). More than 4 billion sequences were read in a total of 30 samples and with a minimum of 15 million sequence depth per sample (Figure S2). In case of differential gene expression study, the transcripts that were passed through p value significance (< 0.05) and FC (> 1.5) filters were considered as significant changes in gene expression from a total of > 40,000 transcripts expressed in PHHs.

### Pathway enrichment analysis

Pathway enrichment data analysis was performed using Ingenuity Pathway Analysis or IPA (Qiagen Inc., https://www.qiagenbioinformatics.com/products/ingenuitypathway-Analysis) according to the standard protocols as previously reported^50^. In this, we overlaid a list of rifamycin RGs on more than 500 cellular and metabolic pathways available in IPA to identify various hepatic and metabolic pathways regulated during rifampin, rifabutin and rifapentine treatments in PHHs as previously described^51^. UNMC has a license to IPA software and subscribers can use it to generate images of pathways and gene networks that can be published without any consent from Qiagen. IPA provided a list of regulated pathways based on the involvement of differentially expressed transcripts from a particular dataset. ‘Core analysis’ was performed on the list of significantly up and down regulated differentially expressed drug responsive genes (0.05p and 1.5 FC) to interpret biological pathways according to the standard protocol^51^. We set several parameters, including, ‘p value’ significance, ‘ratio’ of total number of genes involved in a pathway regulated by RRGs to a total number of genes known in a pathway and ‘z score’ that strongly predicts significantly regulated pathways. Pathways that were passed through the filters of < 0.01 *p* value significance, 0.1 ratio that constitutes to a minimum involvement of 10 percent of differentially expressed genes in a particular pathway to a total number of pathway specific genes and positive activation z score of 2.0 that strongly predict the influence on a pathway were considered as significantly regulated pathways.

### Transcriptional network and upstream regulator analysis (URA)

Transcriptional network and functional analyses were generated through the use of IPA (Qiagen Inc., (Qiagen Inc., https://www.qiagenbioinformatics.com/products/ingenuitypathway-Analysis) according to the standard protocols as previously reported^50^. In a IPA core analysis of rifampin, rifabutin and rifapentine RGs, we compared rifamycin RG data sets to find a link to each of the drug responsive network on the commonly shared genes. Genes unrelated to metabolic pathways were removed from our analysis. Metabolic networks containing DMEs, drug transporters and TFs were separately analyzed. TFs involved in regulation of transcriptional network operated genes were identified and their significance and targets were identified by URA. URA of transcripts was performed to identify significantly (*p* = 0.01) regulated TFs either in the list of rifamycin RGs or based on prediction. TFs with significant Z score value (> 2.0) were predicted as activated or inhibited pathways based on the prediction of IPA software.

### Statistical analysis

Statistically significant differences in rifampin, rifabutin and rifapentine ICC in PHHs was performed between two groups using a student t test in Prism sofware (Graph Pad Sofware Inc, La Jolla, USA). A difference with > 0.05 *p* value between the two groups was considered significant. Significance analyses in differential gene expression, pathway and upstream regulator analyses were described in their respective sections above.

## Supplementary information

Supplementary file1 (DOCX 1307 kb)
