# Supplementary material for: Hepatocytic transcriptional signatures predict comparative drug interaction potential of rifamycin antibiotics
Source: Sci Rep. 2020 Jul 28;10:12565. doi: 10.1038/s41598-020-69228-z (PMC7387492; doi:10.1038/s41598-020-69228-z)
Supplement: Supplementary file 1 — Supplementary file1 (DOCX 1307 kb) [file 41598_2020_69228_MOESM1_ESM.docx]

**Supplementary Information**

**Hepatocytic transcriptional signatures predict comparative drug interaction potential of rifamycin antibiotics**

Shetty Ravi Dyavar^a^*, Timothy M. Mykris^a^, Lee C. Winchester^a^, Kimberly K. Scarsi^a^, Courtney V. Fletcher^a^, Anthony T. Podany^a^*

**^a^** Antiviral Pharmacology Laboratory, University of Nebraska Medical Center (UNMC) Center for Drug Discovery, UNMC, Omaha, NE 68198 USA

***Correspondence:** [shettyravi.dyavar@unmc.edu](mailto:shettyravi.dyavar@unmc.edu) and [apodany@unmc.edu](mailto:apodany@unmc.edu)

**Supplementary Figures**

**
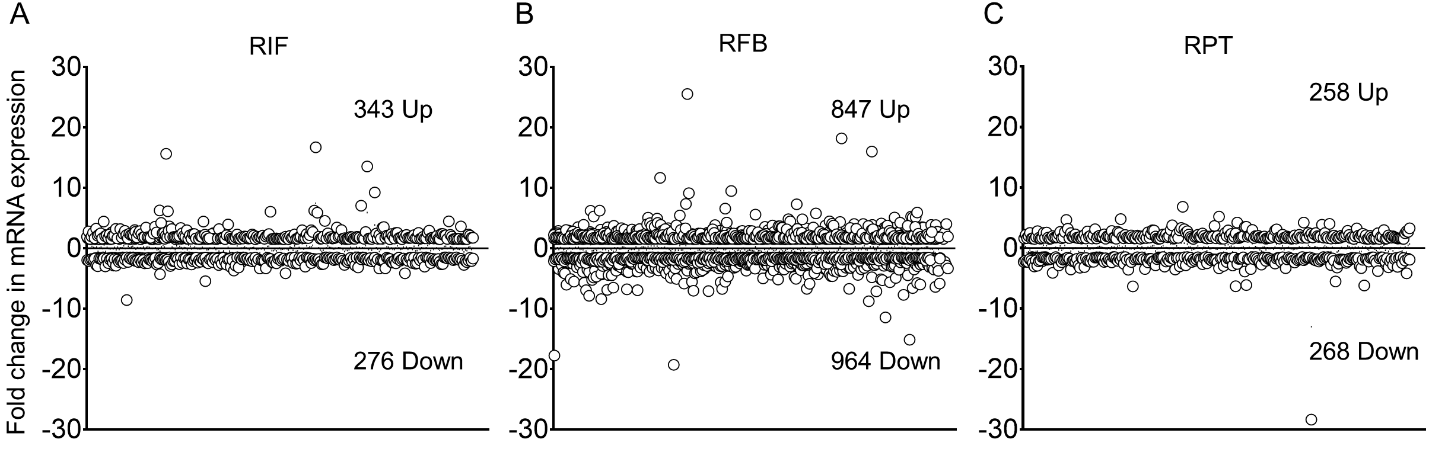
**

**Figure S1. Transcripts differentially expressed in PHHs with treatment of rifampin (RIF), rifabutin (RFB) and rifapentine (RPT).** Transcripts expressed in response to 2A) RIF, 2B) RFB and 2C) RPT treatment in PHHs are shown. Transcripts with a minimum 0.05 p value and 1.5 fold change or FC are considered significant and are shown.

**
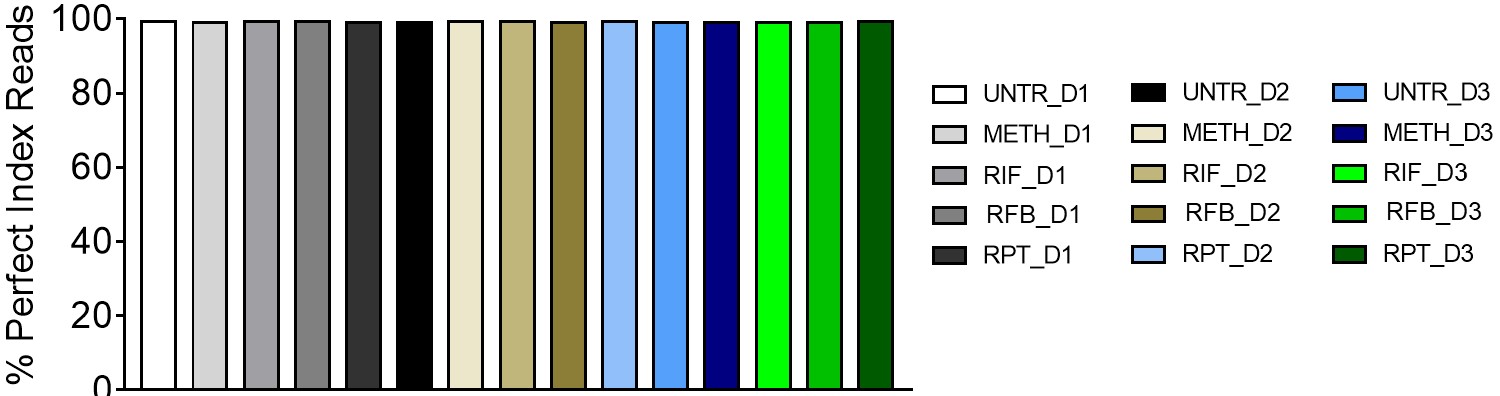
**

**Figure S2.** Qualitative analysis of RNA sequencing reads. Perfect Index Reads (PIR) of RNA sequences obtained by performing next generation sequencing (NGS) of mRNA extracted from rifampin (RIF), rifabutin (RFB) and rifapentine (RPT) treated PHHs are shown.

**
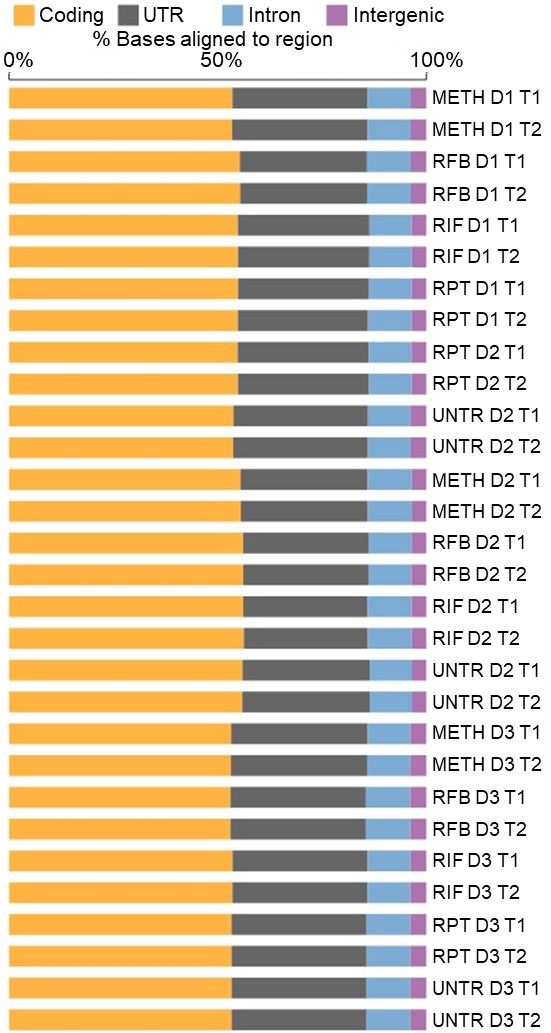
**

**Figure S3**. RNA sequences obtained by performing next generation sequencing (NGS) of mRNA extracted from rifampin (RIF), rifabutin (RFB) and rifapentine (RPT) drug treated PHHs were aligned based on various regions of expressed transcripts sequenced with Illumina HiSeq2500 (Illumina Inc, USA). Percentage of coding (yellow), untranslated region or UTR associated (grey), intronic (blue) and intergenic (violet) bases in technical replicates of RIF, RFB and RPT samples are shown.

**
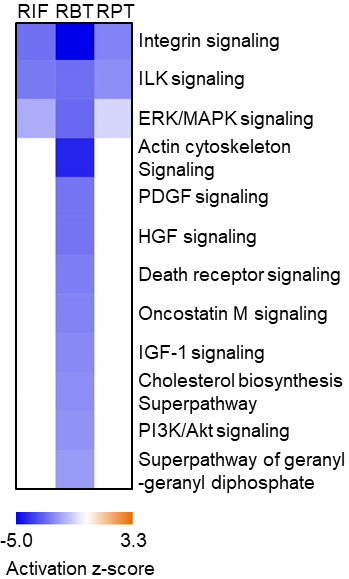
**

**Figure S4. Pathways inhibited by rifamycin antibiotics in PHHs.** Core analysis of a list of down regulated genes (0.05 p, 1.5 FC) in response to rifampin (RIF), rifabutin (RFB) and rifapentine (RPT) treatments in PHHs was performed by ingenuity pathway analysis (IPA) software. Pathways significantly (<0.01 p, >0.1 ratio and >2.0 z score) regulated in response to RIF, RFB and RPT treatments in PHHs were identified by pathway enrichment analysis.

**Supplementary Tables and table legends**

Table S1. Demographic details of three healthy donors of hepatocytes.

| **Donor Information** | **HU8210** | **HUM4079** | **HUM4119B** |
| --- | --- | --- | --- |
| Gender | Male | Male | Female |
| Race | Caucacian | Caucacian | African American |
| Age | 51 | 24 | 30 |
| BMI | 22 | 31.5 | 33 |
| Tobacco Use | No | No | No |
| Alcohol Consumption | Rare | Rare | No |
| Drug use | No | No | No |
| Hepatitis B | -ve | -ve | -ve |
| Hepatitis C | -ve | -ve | -ve |
| HIV | -ve | -ve | -ve |
| Viability | >88% | >86% | >97% |
| Source | Invitrogen, USA | Triangle research Labs, USA | Triangle research Labs, USA |

Abbreviations: BMI, body mass index and HIV, human immunodeficiency virus.





Table S3. Pathways significantly (0.01p value, 0.1 ratio and 2.0 z score) up regulated by multiple rifamycin antibiotics in primary human hepatocytes:

| **Pathway** | **Pathway specific genes induced in response to the treatment** | | | | | | | |
| --- | --- | --- | --- | --- | --- | --- | --- | --- |
|  | **RIF** | **p-value** | **RFB** | **p-value** | **RPT** | **p-value** | **RIF & RFB** | **RIF, RFB & RPT** |
| Acetone degradation I | - | 1.26E-07 | CYP2C19 | 4.37E-06 | CYP2S1 | 2.14E-04 | CYP3A5, 2C9, 2A6, 2B6, 2C8 | CYP3A7, 3A4, 2C8 |
| Bupropion degradation | - | 3.16E-08 | CYP2C19 | 9.33E-07 | CYP2S1 | 1.02E-04 | CYP3A5, 2C9, 2A6, 2B6 | CYP3A7, 3A4, 2C8 |
| Estrogen biosynthesis | - | 1.23E-06 | CYP2C19, HSD17B14 | 6.31E-06 | CYP2S1,  HSD17B13 | 5.13E-05 | CYP3A5, 2C9, 2A6, 2B6 | CYP3A7, 3A4, 2C8 |
| Melatonin degradation  superpathway | UGT2B4,  2B17, B15, | 2.51E-14 | CYP2C19,  MAOB | 6.17E-08 | UGT1A7,  CYP2S1 | 4.57E-07 | CYP2A6, 2B6, 2C9, 3A5; UGT1A1, 1A3 | CYP2C8, 3A4, 3A7; UGT1A4, 1A5;SULT2A1 |
| Melatonin degradation-I | UGT2B4,  2B17, 2B15 | 7.94E-15 | CYP2C19 | 1.82E-07 | UGT1A7,  CYP2S1 | 2.57E-07 | CYP2A6, 2B6, 2C9, 3A5; UGT1A1, 1A3 | CYP2C8, 3A4, 3A7; UGT1A4, 1A5;SULT2A1 |
| Nicotine degradation-II | UGT2B4,  2B17, 2B15 | 2.00E-13 | CYP2C19 | 1.35E-06 | UGT1A7,  CYP2S1 | 3.72E-06 | CYP2A6, 2B6, 2C9, 3A5; UGT1A1, 1A3 | CYP2C8, 3A4, 3A7; UGT1A4, 1A5 |
| Nicotine degradation-III | UGT2B4,  2B17, 2B15 | 2.00E-14 | CYP2C19 | 2.45E-07 | UGT1A7,  CYP2S1 | 1.32E-06 | CYP2A6, 2B6, 2C9, 3A5; UGT1A1, 1A3 | CYP2C8, 3A4, 3A7; UGT1A4, 1A5 |
| Metabolic Pathways up regulated by both Rifampin and Rifabutin | | | | | | | | |
| Thyroid hormone metabolism-II | UGT2B4, 2B17, 2B15 | 5.37E-09 | - | 2.82E-03 | **-** | - | UGT1A1, 1A3, A4, 1A5;DIO1, SULT2A1 |  |

Table S4. Pathways significantly (0.01p value, 0.1 ratio and 2.0 z score) up regulated by rifampin and rifabutin in primary human hepatocytes:

| **Pathway** | **P value** | **Induced genes** |
| --- | --- | --- |
| Pathways up regulated by Rifampin: | | |
| Serotonin degradation | 1.00E-05 | UGT1A1, 1A3, 1A4, 2B4, 2B15, 2B17, DIO1, SULT2A1 |
| Pathways up regulated by Rifabutin: | | |
| Complement System | 1.29E-03 | C4A/C4B, MBL2, C1S, MASP1, C2, C5 |
| LXR/RXR Activation | 5.01E-05 | ABCG5 & 8, KNG1, APO B & E, SERPINF1, UGT1A3, PCYOX1, C4A/C4B, ORM1 & 2, CYP7A1, CD14, TLR3 |
| Bile Acid Biosynthesis Pathway | 4.17E-05 | AMACR, SLC27A5, CYP3A4, AKR1D1, CYP7A1 |
| Glycine betaine degradation | 2.19E-04 | SARDH, BHMT, DMGDH, PIPOX |
| Methionine Degradation | 1.29E-03 | CBS/CBSL, BHMT, MAT1A, MCEE, CDO1, CTH |

Major abbreviations of rifamycin drugs and enzyme classes: RIF, rifampin; RFB, rifabutin; RPT, rifapentine; CYP, cytochrome P450; UGT, UDP glucuronosyltransferase; MAOB, monoamine oxidase; HSD, Hydroxysteroid dehydrogenase; DIO1, Iodothyronine deiodinase 1 and SULT, sulfotransferase.

Table S5. Pathways significantly (0.01p value, 0.1 ratio and 2.0 z score) down regulated by rifabutin in primary human hepatocytes:

| **Pathway** | **P value** | **Induced genes** |
| --- | --- | --- |
| Integrin signaling | 1.0E-10 | RAC2, PFN1, ITGA10, BCAR1, RHOH, SHC1, ITGA3, CAV1, ITGAV, IRS2, ITGB4, VCL, ACTA1, ACTN1, VASP, CAPN6, PXN, ASAP1, ACTB, ITGA2, RALB, ITGA5, TSPAN2, PDGFB, CAPN8, WIPF1, RND3, TSPAN1, ZYX, PIK3CD, NEDD9, ITGA7, ACTG1 |
| Actin cytoskeleton signaling | 1.20E-06 | RAC2, PFN1, F2R, TRIO, IQGAP1, BCAR1, LIMK1, SHC1, ITGA3, EZR, IRS2, VCL, GNA13, ACTA1, ACTN1, IQGAP3, PXN, TIAM1, ACTB, RALB, ITGA2, ITGA5, PDGFB, TIAM2, PIK3CD, ACTG1, MSN |
| IGF-1 signaling | 4.79E-03 | SOCS1, PXN, YWHAG, CTGF, YWHAH, RALB, YWHAZ, SHC1, FOS, JUN, IGFBP3, IGF1R, PIK3CD, IRS2, IGFBP1, SFN, CYR61 |
| ILK signaling | 1.15E-05 | FLNB, PXN, ACTB, BMP2, SNAI1, FERMT2, VIM, HIF1A, CREB5, RHOH, FOS, JUN, RND3, FLNC, PIK3CD, IRS2, PPP2R2C, KRT18, ITGB4, VCL, ACTG1, ACTA1, ACTN1 |
| ERK/MAPK signaling | 3.72E-04 | ETS1, PPARG, RAC2, PPP1R14C, PXN, YWHAG, YWHAH, RALB, ITGA2, YWHAZ, ITGA5, CREB5, BCAR1, SHC1, ELF4, FOS, ITGA3, ETS2, DUSP4, PIK3CD, IRS2, PPP2R2C, HSPB1 |
| Regulation of cellular mechanics by calpain | 3.09E-06 | CAPN8, PXN, CAPN6, ITGA3, EZR, ITGA2, RALB, ITGA5, VCL, ACTN1 |
| PI3K/AKT signaling | 6.46E-06 | YWHAG, YWHAH, GDF15, ITGA2, RALB, YWHAZ, ITGA5, NOS3, SHC1, BCL2L1, ITGA3, CDKN1A, PPP2R2C, PIK3CD, SFN |
| HGF signaling | 1.00E-10 | ETS1, ELF4, FOS, PXN, ITGA3, JUN, MAP3K6, ETS2, CDKN1A, RALB, ITGA2, ITGA5, PIK3CD, IRS2 |
| Oncostatin M signaling | 1.07E-03 | MT2A, SHC1, EPAS1, RALB, OSMR, PLAU, MMP1 |
| PDGF signaling | 1.91E-03 | SHC1, FOS, JUN, ABL2, RALB, SPHK1, PDGFRA, CAV1, PIK3CD, IRS2, PDGFB |
| Geranylgeranyldiphosphate biosynthesis I | 3.09E-04 | MVD, FDPS, IDI1, HMGCS1 |
| Death receptor signaling | 2.24E-04 | TNFRSF21, ACTB, LMNA, ACTG1, TNFRSF10A, ARHGDIB, ACTA1, FASLG, LIMK1, HSPB1 |

Table S6. Transcription factors (TFs) predicted to control the expression of target gens induced during rifampin (RIF), rifabutin (RFB) and rifapentine (RPT) treatment in hepatocytes that further regulate drug metabolism networks were identified by upstream regulator analysis tool.

| **TFs** | **Rifampin**  **(RIF)** | **Rifabutin (RFB)** | **Rifapentine (RPT)** | **RIF & RFB** | **RFB**  **&**  **RPT** | **RIF & RPT** | **RIF, RFB & RPT** |
| --- | --- | --- | --- | --- | --- | --- | --- |
| **FOXA3** |  | ACADM,CYP2C19, PCK1 |  | CYP2C9, 3A5; FOXA1 | BHMT | AKR1B1 | CYP2C8, 3A4, 3A7 |
| **HNF4α** | ACTA2, ASPRV1, CACNA2D2, CHEK2, CHI3L1, EHD3, GPX2, NUAK1, PROM1, PTK7, RASL12, SPATA6, SPP1, UGT2B15, USP2 | A1CF, ABCC6 & G8; ACY3, ALDH5A1, 8A1; AMACR, ANG, ANKRA2, ANXA9, APOB & E, AQP9, ARG1, AS3MT, BACE1, BCKDHA, BET1, BST1, C11orf54, C2, CBS/CBSL, CD1D, CDK2AP2, CYP2C9, 7A1, 7B1, DNAJB9, EFNA1, EMC9, F7, FDXR, FUZ, GGCX, GLYAT, HAAO, HIST1H2BD, HSP90B1 & A5, HUNK, ING4, ITGAL, KBTBD7, KCNN2, KNG1, LINC00574, MAOB, MST1, MTHFS, NBR1, OAZ2, ORM1, ORM2, PAN2, PARD3B, PARP9, PCK1 & 2, PDCD4, PDK2, PLA2G12B, PPARA, PRR3, PSAT1, SAP30L, SAT2, SEC11C, SEC24D, SEL1L, SEM1, SERPINA3 & A4, SHBG, SLC17A2, 33A1, 43A1, SSR2, TAPBPL, TESK2, TTC38, VN1R1, YPEL3 | ARHGEF19,  CCNA2,  HIST1H2AD,  HMGB2,  KIF20A,  KRT7,  PPP1R3B & C; SAA1 & 2;  SEM1,  TUBB4A,  ZBTB37,  ZNF71 | ABCG5, BDH1, C1S, C4A/C4B, CCL16, COQ8A, CYP2B6, 2C9; FCAMR, FETUB, FOXA1, GADD45G, GPC6, MCEE, PRODH2, SERPINA5, UGT1A1, VSNL1 | CYP11A1,  HAO2, KCNC3, KLF15 |  | CCDC170, CYP2C8, 3A4, 3A7, 3A43, EPHX1, ETNPPL, F13B,  MBL2,  SLC38A4, SULT2A1 |
| **NR1I2**  **(PXR)** |  | ANG, CES3, CTH, CYP2C19, 4V2, 7A1, ENTPD5, PDIA4, PIPOX, PPARA |  | ABCB1, ALAS1, CYP2B6, 2C9, 3A5, UGT1A1 & 1A3 | BHMT |  | CYP2C8, 3A4, 3A7, UGT1A4, SULT2A1, |
| **NR1I3**  **(CAR)** | UGT2B17 | CYP2C19, 7A1, 7B1; PCK1, TMPRSS2 |  | ABCB1, ALAS1, CYP2B6, 2C9, 3A5, DIO1, UGT1A1, 1A3 | RARRES1 |  | CYP2C8, CYP3A4, CYP3A7, EPHX1,  SULT2A1, |
| **NR3C1** | EDN2, GLP2R, PDE4B, PDPN, PIGR, SCNN1A, SLC2A5, SPP1, | APOE, AS3MT, BCKDHA, BMF, BRCA1, CYP11A1, 2C19, 7A1; DNASE1L3, EFNA1, HERPUD1, IFIH1, IGFALS, IRF1, KLF9, LMO4, MAT1A, PCK1, PDCD4, PIGR, PPARA, SELE, SLC43A1, TSC22D3 | CD83,  CXCL10,  CYP11A1,  GHRHR,  GLP2R,  IP6K3,  TK1 | ARRB1, C4A/C4B, COQ8A, CYP2C9, CYP3A5, SERPINA6, TLR5 |  | CCL2, | APOL3, CYP2C8, 3A4, 3A7; TLR1, TNFSF4 & 14 |
| **RXRα** | ALPI,  MYL7,  NPPB,  SPP1,  STRA6 | ACADM, APOE, BRCA1, CYP7A1, KLF9, KNG1, MAOB, PCK1 & 2, PLIN1, PPARA, SLC27A5 | CCNA2,  CXCL10,  GHRHR,  TK1 | ABCB1, ABCB4, CDO1, CRYAA/  CRYAA2, GCK, CYP2B6,2C9, 3A5; FOXA1, SLC51B |  |  | CYP3A4,  EPHX1,  SULT2A1 |
